# Low-Level Ozone Exposure and Respiratory Symptoms in Infants

**DOI:** 10.1289/ehp.8559

**Published:** 2005-12-29

**Authors:** Elizabeth W. Triche, Janneane F. Gent, Theodore R. Holford, Kathleen Belanger, Michael B. Bracken, William S. Beckett, Luke Naeher, Jean-ellen McSharry, Brian P. Leaderer

**Affiliations:** 1 Yale Center for Perinatal, Pediatric, and Environmental Epidemiology, Department of Epidemiology and Public Health and; 2 Department of Obstetrics and Gynecology, Yale University School of Medicine, New Haven, Connecticut, USA; 3 Departments of Environmental Medicine and Medicine, University of Rochester School of Medicine and Dentistry, Rochester, New York, USA; 4 Department of Environmental Health Science, College of Public Health, University of Georgia, Athens, Georgia, USA

**Keywords:** air pollution, difficulty breathing, infant symptoms, maternal asthma, wheeze

## Abstract

**Objective:**

Recent studies indicate that the U.S. Environmental Protection Agency (EPA) ozone standards may not protect sensitive individuals. In this study we examined respiratory effects of ozone in infants who may be vulnerable, particularly if they are children of asthmatic mothers.

**Design:**

Women delivering babies at one of five hospitals in southwestern Virginia between 1994 and 1996 were invited to participate in a cohort study; 780 women enrolled. Ambient air quality data (ozone and particulate matter) were collected at a central monitoring site.

**Participants:**

This analysis is of 691 infants followed for approximately 83 days between 10 June and 31 August 1995 and/or 1996; they contributed a total of 52,421 infant-days of follow-up. Mothers were interviewed at enrollment and approximately biweekly to report infants’ daily symptoms. Repeated measures logistic regression models were run separately for wheeze, difficulty breathing, and cough. Ozone metrics included 24-hr average, peak 1-hr, and maximum 8-hr average. Analyses were repeated for the 61 infants whose mothers had asthma.

**Results:**

For every interquartile-range increase in same-day 24-hr average ozone, likelihood of wheeze increased 37% [95% confidence interval (CI), 2–84%]. Among infants of asthmatic mothers, same-day 24-hr average ozone increased likelihood of wheeze 59% (95% CI, 1–154%) and of difficulty breathing 83% (95% CI, 42–136%). Maximum 8-hr ozone and peak 1-hr ozone were associated with difficulty breathing, but not wheeze, in infants of asthmatic mothers. Ozone was not associated with cough.

**Conclusions:**

At levels of ozone exposure near or below current U.S. EPA standards, infants are at increased risk of respiratory symptoms, particularly infants whose mothers have physician-diagnosed asthma.

Ozone is a common summertime pollutant formed by reactions of ambient nitrogen oxides and volatile organic compounds (VOCs) in the presence of sunlight and heat. Animal and human studies have identified specific effects O_3_ has on the respiratory system, including alterations in airway epithelium (Bromberg et al. 1991; Devalia et al. 1996; Sandstrom et al. 1991; Wagner et al. 2001), airway hyperresponsiveness (Devalia et al. 1996), airway infiltration by inflammatory cells (Chitano et al. 1995), and enhancement of antigen-associated airway inflammation (Depuydt et al. 2002). Because O_3_ is a relatively insoluble gas, it tends to pass through the upper respiratory tract and penetrate deep into the lung (Mathieu-Nolf 2002).

O_3_ has consistently been linked to acute respiratory effects and to hospital admissions in human populations living in highly polluted areas (Ostro et al. 1995, 2001; Romieu et al. 1996, 1997; Thurston et al. 1992). U.S. Environmental Protection Agency (EPA) standards based on peak 1-hr O_3_ concentrations and maximum 8-hr running averages have been established (U.S. EPA 2006), but recent studies indicate that these standards may not protect sensitive individuals (e.g., children with asthma) from acute respiratory responses (Gent et al. 2003; Mortimer et al. 2000; Thurston et al. 1997).

Young children may be particularly sensitive to O_3_, because significant lung development continues postnatally (Finkelstein and Johnston 2004). Differences in lung anatomy and physiology, ventilation rates, and organ maturity between children and adults may explain children’s greater vulnerability to air pollutants (Mathieu-Nolf 2002). Infants, for whom transient wheeze is common (reported in up to 50% of healthy infants), and infants with a genetic predisposition (e.g., maternal asthma) may be even more sensitive to pollutant effects. There has been a dearth of research examining respiratory health effects of O_3_ in infants who may be at greatest risk of O_3_’s effects. In this study we examined acute respiratory effects of relatively low O_3_ levels in infants living in nonsmoking households.

## Methods

### Study population

A total of 12,128 women delivering babies at one of five participating hospitals in southwestern Virginia between 1994 and 1996 were screened for eligibility into a cohort study investigating the effects of exposure to acid aerosols on infant respiratory health. Exclusion criteria included any smoking in the household, infant death or adoption, maternal age < 19 years, and non-English-speaking respondent. The study was designed to oversample households with exposure to kerosene heaters (22%), the primary indoor source of acid aerosols, and gas stoves (28%). About half of the study families did not use either a gas stove or a kerosene heater at all during the year (e.g., they used an electric stove). A total of 1,331 women were invited to participate, of whom 780 enrolled. During the summers of 1995 and 1996, study equipment was set up at a central site in Virginia to monitor daily levels of air pollutants. The current analysis is limited to the 691 infants who were followed between 10 June and 31 August 1995, and/or 10 June and 31 August 1996. None of the homes used kerosene heaters during this study period.

The study was reviewed and approved by the Yale Human Investigations Committee as well as the institutional review boards of each of the participating hospitals. A standardized questionnaire was administered to mothers of study infants at enrollment (when the infant was 3–5 months of age) by a trained research assistant. Informed consent was obtained from the mother. The initial questionnaire gathered detailed information on the health history of the infant, household demographic data, maternal health history, and dwelling characteristics.

### Outcome assessment: infant respiratory symptoms

At the initial visit, mothers were given a calendar on which to record infants’ daily respiratory symptoms, including wheeze, difficulty breathing, and cough. At approximately biweekly intervals [median = 16 days; interquartile range (IQR) = 13–19 days] for 1 year, a research assistant called the mother, who reported the presence or absence of each symptom on each day during the reporting period.

### Exposure assessment: ambient air pollution and meteorology

Ambient air quality data were collected at a central monitoring site in Vinton, Virginia (greater Roanoke area). Hourly O_3_ was collected by the Department of Environmental Quality (DEQ) (Richmond, VA) at this site and was summarized into *a*) 24-hr average, *b*) peak 1-hr concentration, and *c*) maximum 8-hr running average. During the summers of 1995 and 1996, integrated 24-hr particulate mass—including particulate matter < 2.5 μm in diameter (PM_2.5_) and particulate matter < 10 μm in diameter (PM_10_)—was measured using study equipment set up adjacent to the DEQ monitoring site. Harvard-Marple impactors (Air Diagnostics, Harrison, ME) collected PM_2.5_ and PM_10_ samples at 10 and 4 L/min, respectively. Coarse particle (2.5 < aerodynamic diameter < 10 μm) concentrations were calculated as the difference between PM_10_ and PM_2.5_ concentrations. Hourly meteorological data were collected from Roanoke Airport, located 6 miles from the central monitoring site. The monitoring sites were located within 115 miles of all five hospitals from which subjects were recruited.

### Statistical analysis

Each infant was followed for 1 year, including 83 days during the summer (10 June–31 August) of 1995 and/or 1996. This analysis is limited to the summer period. The 691 infants who were followed in either of the summers contributed a total of 52,421 (91%) infant-days of follow-up out of a possible 57,353 days (691 infants × 83 days). A total of 61 infants (9%) whose mothers had a history of physician-diagnosed asthma contributed 4,449 infant-days. To exploit the repeated observations for each infant, we performed logistic regression analyses using the GENMOD procedure in SAS (version 8; SAS Institute Inc., Cary, NC) with the AR1 autoregressive correlation structure among repeated observations for the same individual were performed. The GENMOD procedure uses the generalized estimating equations (GEE) method for fitting generalized linear models to handle correlated repeated measures data. To determine the appropriate form for the correlation structure among the repeated measures for each individual, we fitted an *m*-dependent (*m* = 14) model that did not assume a particular relationship between correlation and lag. A graph was constructed (not shown), which revealed that log correlation was linearly related to the lag, consistent with an AR1 model, which we used for the analysis presented here. We ran separate models for the following outcomes: wheeze, difficulty breathing, and cough. We examined three different O_3_ metrics separately: 24-hr average, peak 1-hr, and maximum 8-hr running average. Single pollutant and co-pollutant models for each outcome were built. Pollutant measures for same day and previous day were considered. Single pollutant models of O_3_ effects controlled for daytime temperature and humidity, while co-pollutant models controlled for PM_2.5_, coarse particles, temperature, and humidity. The repeated-measures analysis allowed each subject to serve as his or her own control so personal variables, except for age, were not included in the models. Analyses were conducted for the entire population of infants (*n* = 691), and then repeated for the subset of infants whose mothers had asthma (*n* = 61).

## Results

As expected, approximately half the study infants were boys, and > 40% were the only child in the household (Table 1). Mothers of the infants tended to be married (80%) and white, non-Hispanic (75%). More than one-third of the mothers had high school or less education, while another 36% had college or higher. There were no significant differences in distributions of these characteristics by maternal asthma status, although there appeared to be more pets in homes of asthmatic mothers (51%) than nonasthmatic mothers (42%).

Table 2 presents distributions of ambient pollutants measured daily during the summers of 1995 and 1996. The mean (± SD) 24-hr average concentration of O_3_ across the 166 days of the study period was 35.2 ± 8.4 ppb. Mean concentration of both maximum 8-hr running average (54.5 ppb) and peak 1 hr (60.8 ppb) for this period were below the U.S. EPA standards for these metrics of 80 ppb and 120 ppb, respectively (U.S. EPA 2006). The mean concentration of PM_2.5_ (23.2 μg/m^3^) was also well below the U.S. EPA standard of 65 μg/m^3^ (U.S. EPA 2004).

**Figure 1:**
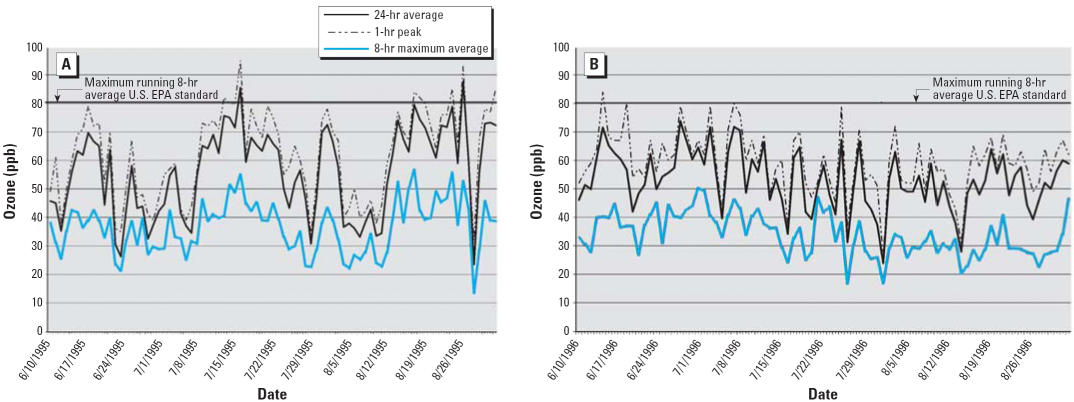
Daily levels of ozone (24-hr average, peak 1-hr, maximum 8-hr average): (*A*) June 10–August 31, 1995; (*B*) June 10–August 31, 1996.

The daily variability in the three different metrics of O_3_ (24-hr average, maximum 8-hr running average, and peak 1-hr average) are shown for 1995 (Figure 1A) and 1996 (Figure 1B). Levels of O_3_ tended to be low during the study period, with maximum 8-hr running average exceeding the U.S. EPA standard of 80 ppb on only 2 days in 1995 and no days in 1996. The 120-ppm 1-hr U.S. EPA standard was not exceeded in either year.

Correlations between same-day and previous-day concentrations of O_3_ were moderate, *r* = 0.45–0.55 (data not shown), and similar correlations were found between O_3_ and PM_2.5_. O_3_ measures were more highly inversely correlated with humidity (*r* = −0.62 to −0.69) than positively correlated with temperature (*r* = 0.26–0.55). Correlations between the three O_3_ metrics ranged from 0.74 (24-hr average with maximum 8-hr running average) to 0.96 (maximum 8-hr running average with peak 1 hr) (data not shown).

Lower respiratory symptoms (wheeze and difficulty breathing) were infrequent in these infants during the summer study period (Table 3). For example only 8% of the 691 infants wheezed at least once during this period, and of the 52,421 infant days of follow-up contributed by the 691 infants, there were 310 (0.6%) wheeze days. Infants whose mothers had asthma were more likely to have each of these symptoms than infants whose mothers did not have asthma.

In the single-pollutant GEE models, none of the O_3_ measures were associated with any respiratory symptom among the total study population of infants (Table 4, top). Among the subset of infants whose mothers had asthma (Table 4, bottom), same-day 24-hr average O_3_ was more strongly and consistently associated with lower respiratory symptoms than either maximum running 8-hr average or peak 1-hr O_3_. Same-day 24-hr average O_3_ was statistically significantly associated with wheeze [odds ratio (OR) = 1.65; 95% confidence interval (CI), 1.01–2.70 per IQR increase in O_3_] and difficulty breathing (OR = 2.14; 95% CI, 1.42–3.20). Previous-day 24-hr average O_3_ also approached statistical significance with difficulty breathing (OR = 1.49; 95% CI, 0.96–2.32). Same-day maximum running 8-hr average O_3_ (OR = 1.67; 95% CI, 1.02–2.73) was significantly associated with difficulty breathing but not wheeze, and peak 1-hr O_3_ was marginally associated with difficulty breathing (OR = 1.64; 95% CI, 0.95–2.83). None of the O_3_ measures were associated with cough (data for cough not shown).

Three co-pollutant models were run for each outcome and each O_3_ metric: model 1, same-day pollutant measurements (O_3_, PM_2.5_, and coarse particles); model 2, previous-day pollutant measures; model 3, same-day and previous-day pollutant measurements.

In co-pollutant models for all study infants (Table 5, top), same-day 24-hr average O_3_ was associated with wheeze in model 1 (OR = 1.41; 95% CI, 1.03–1.93) controlling for same- day PM_2.5_, coarse particles, average temperature, humidity, and infant age at the beginning of follow-up, and in model 3 (OR = 1.37; 95% CI, 1.02–1.84) controlling for same-day and previous-day exposures. Same-day maximum 8-hr running average O_3_ was marginally associated with difficulty breathing in these models. None of the O_3_ metrics were associated with cough (data for cough not shown).

In co-pollutant models of the subset of infants whose mothers had asthma, same-day 24-hr average O_3_ remained significantly related to wheeze and difficulty breathing (Table 5, bottom, models 1 and 3). In addition, previous-day 24-hr average O_3_ was associated with difficulty breathing (models 2 and 3). Maximum 8-hr O_3_ and peak 1-hr O_3_ were significantly associated with difficulty breathing, but not wheeze or cough. Effect estimates for these two measures of O_3_ were generally lower than those for 24-hr average O_3_. None of the O_3_ measures were significantly associated with cough, controlling for co-pollutants.

## Discussion

In this study we examined the effects of relatively low O_3_ levels on acute respiratory symptoms in infants living in nonsmoking households in southwestern Virginia. The results of this study indicate that among the subgroup of infants whose mothers had a history of physician-diagnosed asthma, exposure to O_3_ at levels close to or below current U.S. EPA guidelines increased the likelihood of respiratory symptoms, controlling for PM_2.5_ and coarse particle exposure. The association between same-day 24-hr average O_3_ and wheeze, controlling for particle exposure, was weaker but significant for the overall group of infants in the study.

Earlier U.S. EPA standards were based on peak 1-hr O_3_ exposures (120 ppb), but the standards were revised in 1997 after the 1-hr standard was determined to be inadequate for protecting human health (U.S. EPA 2006). The new standard, based on maximum running 8-hr average, is 80 ppb. In our study, there were no days on which the earlier peak 1-hr standard was exceeded in either 1995 or 1996, and only 2 days on which revised maximum 8-hr average O_3_ standard was exceeded (both in 1995). Although associations were found between each of these metrics and respiratory symptoms in the subgroup of infants whose mothers had asthma, our findings suggest that 24-hr average O_3_ was more consistently and strongly associated with acute respiratory symptoms than the standard metrics (peak 1-hr O_3_ and maximum 8-hr average) in these infants.

Several strengths of this study are worth noting. First, this was one of the first studies examining effects of O_3_ exposure on infants of mothers with asthma, a potentially vulnerable subpopulation. Although asthma is not typically diagnosed in the first year of life, wheezing is fairly common in all infants, most likely a consequence of the small size of infant airways and incomplete stage of lung development at birth (Finkelstein and Johnston 2004; Mathieu-Nolf 2002). Thus, all infants may be vulnerable to the effects of air pollutants. The findings of this study suggest that we may be able to identify at birth children who are at particular risk of O_3_-related respiratory effects, namely, those born to mothers who have asthma.

Second, our analytical approach using GEE exploited the repeated nature of the exposure assessment. The approach allows for greater statistical power with a smaller number of subjects while accounting for autocorrelation between repeated observations. We also considered both single-pollutant and co-pollutant (PM_2.5_ and coarse particles) models and controlled for temperature and humidity in all models. Although we presented only the findings for same-day and previous-day exposures, we also examined 3-day and 5-day average O_3_ exposures with similar results: 24-hr average O_3_ was more strongly associated with respiratory symptoms than peak 1-hr O_3_ or maximum 8-hr running average O_3_ measures.

Third, we considered various metrics of O_3_ exposure, including 24-hr average, peak 1-hr O_3_, and maximum 8-hr running average. Different studies have used different metrics of O_3_ exposure, perhaps accounting for some of the inconsistency in findings. By systematically examining all three metrics, despite the fact that they were strongly correlated, our findings indicate that 24-hr average may be more relevant to respiratory symptoms than either of the two metrics on which U.S. EPA standards are based.

Finally, another important strength of the study was the exclusion of any infants with smoking in their household. Considerable evidence suggests that environmental tobacco smoke (ETS) is associated with respiratory symptoms in children (Harlap and Davies 1974; Rantakallio 1978). By including only nonsmoking households, we avoided residual confounding by ETS exposure.

There were some study limitations. Only a small number of infants had asthmatic mothers (*n* = 61), limiting the power of the study to detect small but important effects. However, infants were followed for up to 83 days each, and the analysis allowed them to serve as their own controls. Furthermore, we were able to detect significant associations in this subgroup, indicating that power was not an issue for many associations of interest.

In addition, although we controlled for particle exposure, there may be additional confounders or co-pollutants that were not accounted for in this analysis (e.g., sulfur dioxide and nitrogen dioxide). Although these concentrations were available from central sites, we did not include them in our final models, because they tend to be local rather than regional pollutants. However, we found no differences in our O_3_ findings when central site SO_2_ and NO_2_ concentrations were included (data not shown). Nonetheless, it is possible that other co-pollutants or combinations of pollutants not accounted for in these analyses may partially explain our O_3_ findings.

The analysis was limited to infants from Virginia, and the results may not be generaliz-able to other areas in which the mix or sources of ambient pollutants differ. Importantly, our findings replicate those for our older asthmatic cohort in Connecticut (Gent et al. 2003), an area where comparable O_3_ concentrations were recorded (1-hr and 8-hr averages were 58.6 ppb and 55.5 ppb for 1-hr levels and 51.3 ppb and 50.0 ppb for 8-hr levels). PM_2.5_ levels in the Connecticut study (mean = 13.1 μg/m^3^; median = 10.3 μg/m^3^), however, were approximately half those measured in this study.

Another potential study limitation was that measures of exposure were based on central site rather than personal exposure measurements. However, in a prior study (Gent et al. 2003), we found that levels of ambient PM_2.5_ and O_3_ tended to be regional, with median correlations between sites located throughout Connecticut of 0.91 for particles and 0.81 for O_3_.

Symptoms were reported by mothers of study infants; we did not receive physician confirmation of wheeze or difficulty breathing. It is possible that asthmatic mothers were more likely to recognize symptoms in their infants because of their own personal experience and thus might be more likely to report asthma symptoms in their infants than would nonasthmatic mothers. However, the overall correlation between mother’s and infant’s symptom-days was only 0.05, indicating that mother’s wheeze experiences during the study were unrelated to reporting of wheeze in her infant. In addition, physician confirmation depends mainly on the mother bringing the child in for a visit, which would also be based on the mother’s assumptions about whether her child was wheezing, as well as access to medical care. Thus, using medical chart review for confirmation of symptoms does not necessarily remove bias.

Our finding that O_3_ exposure at these levels was inconsistently related to acute respiratory symptoms in the general cohort of infants is in agreement with several studies failing to find an association between O_3_ and symptoms in healthy, primarily school-age children (Cuijpers et al. 1994, 1995; Linn et al. 1996; Steerenberg et al. 2003). However, four studies did find significant associations between O_3_ and respiratory symptoms (Berry et al. 1991; Castillejos et al. 1992; Schlink et al. 2002; Schwartz et al. 1994). Two of these studies found effects of O_3_ only above defined threshold levels: 30 ppb (Schlink et al. 2002) and 120 ppb (Berry et al. 1991). Another of the studies was in Mexico City (Castillejos et al.1992), where O_3_ concentrations regularly exceed U.S. EPA standards (U.S. EPA 2006). The fourth study (Schwartz et al. 1994) of 300 schoolchildren from six cities found O_3_ related to cough, but only single-pollutant models were analyzed. An additional study (Ward et al. 2002) found inconsistent associations between 24-hr average O_3_ and symptoms among 162 inner-city children 9 years of age.

In contrast, we found that infants whose mothers had asthma were at consistently increased risk of respiratory symptoms with increasing O_3_ exposure. It appears that this subgroup is particularly susceptible to the effects of O_3_. According to the U.S. Clean Air Act (1990), air quality standards must be set low enough to protect susceptible subgroups of the population. Our finding extends those of several studies (Moss et al. 2001; Thurston et al. 1997), including our own (Gent et al. 2003), that identify vulnerable subgroups that may not be protected by current standards.

Many (Buchdahl et al. 2000; Delfino et al. 1998; Gent et al. 2003; Gielen et al. 1997; Just et al. 2002; Mortimer et al. 2000; Romieu et al. 1996, 1997; Schlink et al. 2002; Thurston et al. 1997) but not all (Jalaludin et al. 2004; McConnell et al. 1999; Ostro et al. 2001; Ward et al. 2002) studies in children with asthma or allergies have found that O_3_ exposure is significantly associated with respiratory symptoms or medication use in this high-risk group. Differences in these findings may be due to different O_3_ metrics, co-pollutant versus single-pollutant models, different levels of ambient O_3_, or different time periods or seasons. Jalaludin et al. (2004) concluded that O_3_ was not related to respiratory symptoms or medication use in Australian schoolchildren. However, the study covered an 11-month period including the winter, when symptoms tend to be more frequent and O_3_ is at its lowest.

## Conclusion

Our data provided a unique opportunity to examine the influence of O_3_ and particle exposures on respiratory symptoms in infants living in nonsmoking households. This cohort was followed for a long time, with regular (approximately biweekly) reporting of daily symptoms, and ambient co-pollutants were measured and accounted for in analyses. Even at low levels of O_3_ exposure, infants are at significantly increased risk of respiratory symptoms, particularly if their mothers have a history of physician-diagnosed asthma. This may be linked to a genetic predisposition for asthma, but this susceptibility to the effects of air pollution appears to be identifiable as early as the first year of life, long before a diagnosis of asthma can be made in these infants.

## Figures and Tables

**Table 1:** Selected characteristics of the infant study population, southwestern Virginia, 1995–1996 [*n* (%)].

Characteristic	All infants	Mother does not have asthma	Mother has asthma
Infant sex			
Boy	361 (52.2)	324 (51.4)	37 (60.7)
Girl	330 (47.8)	306 (48.6)	24 (39.3)
No. of other children in household			
0	298 (43.1)	274 (43.5)	24 (39.3)
1	250 (36.2)	230 (36.5)	20 (32.8)
≥ 2	143 (20.7)	126 (20.0)	17 (27.9)
Mother’s marital status			
Married or cohabitating	550 (79.6)	499 (79.2)	51 (83.6)
Divorced	21 (3.0)	19 (3.0)	2 (3.3)
Separated	14 (2.0)	12 (1.9)	2 (3.3)
Never married	106 (15.3)	100 (15.9)	6 (9.8)
Mother’s race			
White, non-Hispanic	519 (75.1)	471 (74.8)	48 (78.7)
African American	161 (23.3)	149 (23.6)	12 (19.7)
Hispanic	3 (0.4)	3 (0.5)	0 (0.0)
Asian	6 (0.9)	6 (1.0)	0 (0.0)
Other	2 (0.3)	1 (0.2)	1 (1.6)
Mother’s education			
≤ High school	246 (35.6)	222 (35.2)	24 (39.4)
Some college	202 (29.2)	183 (29.0)	19 (31.2)
College	176 (25.5)	164 (26.0)	12 (19.7)
> College	67 (9.7)	61 (9.7)	6 (9.8)
Pets in home			
No	393 (56.9)	363 (57.6)	30 (49.2)
Yes	298 (43.1)	267 (42.4)	31 (50.8)

**Table 2:** Distribution of pollutants over study period (*n* = 166 days), summers of 1995 and 1996.

Pollutant	Mean ± SD	Median	Range	25th–75th percentile	IQR
24-hr average O_3_ (ppb)	35.2 ± 8.4	35.7	13.5–56.6	28.8–40.6	11.8
8-hr maximum O_3_ (ppb)	54.5 ± 13.0	55.3	23.5–87.6	45.1–64.1	19.0
1-hr peak O_3_ (ppb)	60.8 ± 13.4	60.5	26.0–95.0	52.0–70.0	18.0
PM_2.5_ (μg/m^3^)	23.2 ± 10.3	22.3	3.5–59.6	15.7–29.4	13.7
Coarse (μg/m^3^)	6.2 ± 3.2	5.9	0.0–19.8	4.2–7.8	3.6

**Table 3:** Frequency of respiratory symptoms and infant-days with symptoms in study infants, by maternal asthma status.

	All infants			Mother does not have asthma			Mother has asthma		
Symptom	Percent of infants with symptoms (n = 691)	No. of infant-days with symptoms (n = 52,421)	Symptom rate^a^	Percent of infants with symptoms (n = 630)	No. of infant-days with symptoms (n = 47,972)	Symptom rate^a^	Percent of infants with symptoms (n = 61)	No. of infant-days with symptoms (n = 4,449)	Symptom rate^a^
Wheeze	8.2	310	0.6	7.5	275	0.6	16.4	35	0.8
Difficulty breathing	5.5	188	0.4	5.2	160	0.3	8.2	28	0.6
Wheeze and/or difficulty breathing	10.9	428	0.8	10.2	384	0.8	18.0	44	1.0
Cough	35.9	1,899	3.6	34.4	1,700	3.5	50.8	199	4.5

a Days of symptoms per 100 infant-days of follow-up.

**Table 4:** Single-pollutant models^a^ of associations between O_3_ measures and respiratory symptoms among all infants and among infants whose mothers had asthma [OR (95% CI)].^b^

O_3_ exposure	Wheeze	Difficulty breathing
All infants		
24-hr average		
Same day	1.32 (0.91–1.92)	1.10 (0.69–1.75)
Previous day	1.18 (0.93–1.50)	1.07 (0.75–1.54)
Maximum 8-hr running average		
Same day	1.04 (0.76–1.43)	1.17 (0.77–1.77)
Previous day	1.11 (0.89–1.38)	1.10 (0.79–1.53)
Peak 1-hr		
Same day	1.00 (0.77–1.31)	1.09 (0.78–1.52)
Previous day	1.12 (0.92–1.36)	1.04 (0.77–1.41)
Infants with mothers who have asthma		
24-hr average		
Same day	1.65* (1.01–2.70)	2.14* (1.42–3.20)
Previous day	1.47 (0.78–2.77)	1.49 (0.96–2.32)
Maximum 8-hr running average		
Same day	1.28 (0.64–2.54)	1.67* (1.02–2.73)
Previous day	1.29 (0.70–2.37)	1.49 (0.73–3.06)
Peak 1-hr		
Same day	1.28 (0.72–2.30)	1.64 (0.95–2.83)
Previous day	1.19 (0.67–2.11)	1.39 (0.76–2.56)

a All single-pollutant models control for 24-hr average temperature, humidity, and infant’s age at beginning of summer study period; lag 1 = previous day.

b OR per IQR increase in O_3_: 24-hr average O_3_ = 11.8 ppb; 8-hr maximum O_3_ = 19.0 ppb; 1-hr peak O_3_ = 18.0 ppb.

* *p* < 0.05.

**Table 5:** Co-pollutant models^a^ of associations between O_3_ measures and respiratory symptoms among all infants and among infants whose mothers had asthma, southwestern Virginia, 1995–1996 [OR (95% CI)].^b^

	Wheeze			Difficulty breathing		
O_3_ exposure^c^	Model 1 (same day)	Model 2 (previous day)	Model 3 (same and previous day)	Model 1 (same day)	Model 2 (previous day)	Model 3 (same and previous day)
All infants						
24-hr average						
Same day	1.41* (1.03–1.93)		1.37* (1.02–1.84)	1.28 (0.84–1.93)		1.19 (0.88–1.62)
Previous day		1.24 (0.96–1.59)	1.13 (0.90–1.44)		1.18 (0.83–1.68)	1.12 (0.82–1.52)
Maximum 8-hr running average						
Same day	1.08 (0.81–1.44)		1.03 (0.79–1.34)	1.37 (0.99–1.89)		1.28 (0.96–1.71)
Previous day		1.15 (0.90–1.47)	1.15 (0.94–1.42)		1.23 (0.90–1.68)	1.19 (0.88–1.61)
Peak 1-hr						
Same day	1.03 (0.81–1.32)		0.98 (0.77–1.24)	1.23 (0.95–1.59)		1.18 (0.94–1.49)
Previous day		1.14 (0.92–1.42)	1.15 (0.95–1.40)		1.13 (0.85–1.51)	1.11 (0.43–1.46)
Infants with mothers who have asthma						
24-hr average						
Same day	1.91* (1.24–2.94)		1.59* (1.00–2.54)	2.31* (1.26–3.69)		1.83* (1.42–2.36)
Previous day		1.53 (0.94–2.49)	1.45 (0.83–2.76)		1.66* (1.11–2.47)	1.53* (1.02–2.28)
Maximum 8-hr running average						
Same day	1.62 (0.75–3.49)		1.36 (0.54–3.43)	1.99* (1.33–2.96)		1.55* (1.02–2.35)
Previous day		1.39 (0.84–2.32)	1.51 (0.96–2.36)		1.57* (1.09–2.26)	1.92* (1.24–2.95)
Peak 1-hr						
Same day	1.57 (0.86–2.86)		1.39 (0.71–2.71)	1.96* (1.28–2.99)		1.73* (1.12–2.66)
Previous day		1.25 (0.74–2.13)	1.30 (0.76–2.22)		1.50* (1.19–2.44)	1.70* (1.19–2.43)

a All co-pollutant models control for PM_2.5_, coarse particles, 24-hr average temperature, humidity and infant’s age at beginning of summer study period; lag 1 = previous day.

b OR per IQR increase in O_3_: 24-hr average O_3_ = 11.8 ppb; 8-hr maximum O_3_ = 19.0 ppb; 1-hr peak O_3_ = 18.0 ppb.

c Separate models were run for each O_3_ exposure metric (24-hr average, maximum 8-hr running average, and peak 1-hr O_3_).

* *p* < 0.05.
